# Supplementary figures and images for: Species-specific, multifaceted venom resistance in Monodelphis domestica reveals novel physiological behavior of von Willebrand Factor under flow
Source: bioRxiv. 2025 May 15:2025.01.21.634112. Preprint. [Version 2] doi: 10.1101/2025.01.21.634112 (PMC12132390; doi:10.1101/2025.01.21.634112)

# Supplementary Figure 1

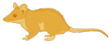

Opossum VWF

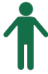

Human VWF

0.24IU

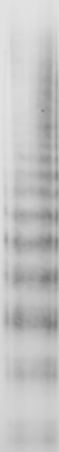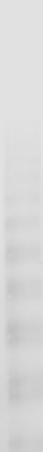

Supplement: Supplementary Figure 1. — Opossum VWF Multimer Gel 1.6% resolving + 0.8% stacking Tris Glycine LDS-SeaKem HGT Agarose Vertical Multimer Gel (8×8cm vertical). Multimeric structures of von Willebrand factor (VWF) were analyzed using a 1.6% lithium dodecyl sulfate (LDS) agarose gel under non-reducing conditions. Lane 2 contained 30 μL of purified Monodelphis domestica VWF (heated at 60°C for 30 minutes) and 10μl loading buffer. Lane 5 contained 30 μL HumateP (human VWF) as a control (heated at 60°C for 30 minutes) and 10μl loading buffer. The gel was transferred to a nitrocellulose membrane and visualized by fluorescence with anti-human VWF antibody (Dako A0082) using a Li-Cor Odyssey CLx Imaging System (Li-Cor). [file media-1.pdf]
